# The Third Molar

**Published:** 1889-01

**Authors:** F. W. Thompson

**Affiliations:** Detroit


### The Third Molar.

BY F. W. THOMPSON, DETROIT.

[Read before the Detroit Dental Society.]

The last erupting tooth of the typical human denture usually makes its appearance at about the 18th year, although the time of its eruption seems less certain than that any of the other teeth. In many cases it is absent, and never makes its appearance. Either the germ is lacking or the peculiar condition of the jaw causing its retention, encysted deep in its process, and its existence revealed only in after years.

The superior third molar is usually very much smaller than the second and its eruption a matter of less pain and disturbance than the lower. They frequently appear turned to the cheek so far as to be useless as masticating organs, and serve only to retain mucous and food between them and the cheek and the second molar, and result in their own and neighbor's destruction, and when in such position, and the other molars are present and normal and any tendency to decay is shown, it would be good practice to remove them at once. It is on the buccal aspect of these teeth that the white, chalky, friable enamel is most frequently found.

The inferior dens sapientia from its position in the thickened and heavy portion of the lower maxilla frequently causes much trouble in making for itself a place in the arch. With all the other teeth anterior it has great difficulty in crowding them forward and in such cases where there is a tendency to short lower jaw the trouble is many times very serious. Cases are known in which the tooth arising partially through the gum and becoming arrested by resistance of the present teeth and ascending ramus have caused extensive ulceration and continuous discharge of pus for a considerable time, and necrosis and purulent infiltration and death have occurred. A case of a student, coming under the care of Drs. Dorrence and Martin at Ann Arbor, the left lower third molar (not carious) had for two years caused pain and annoyance. All the teeth were present in the mouth except the lower wisdom teeth which were evidently trying to get in place. There was a decided tenderness and some swelling one evening, and next morning the patient was unable to open his mouth. Dr. Martin successively applied iodine, warm fomentations, and lanced the part freely, but no effect was obtained. It was finally decided to extract the tooth. The patient was given chloroform, and it was only with great difficulty the tetanized muscles were relaxed, and the mouth opened sufficiently for extraction. Even then the repeated excitation of reflex action by the force employed would close the jaws with great force. After extraction the patient was unable to open his mouth or take solid food for four weeks.

In this tooth, when diseased, the tendency to cause trismus, appears to be peculiar. In the case of Mr. S., Æ. 30; general health fair, a temporary stopping was flown over a slightly exposed pulp. The patient left and was not seen for a week, when he presented himself with a partially ankylosed jaw, and with other symptoms of ulceration. Chloroform was given and the tooth extracted. Large quantities of pus was discharged and much pain suffered for a long time, and it was three months before the mouth could be opened naturally.

Perhaps this tooth has some bearing on the question of "what shall we do with the badly carious first permanent molars of children." It is claimed that if these molars are extracted at the age of 10 to 14, the space will be closed by the moving forward of the second and third molars, and the third molar will develop better qualities than are usually ascribed to it, perhaps owing to the recognized efforts of nature to repair defects.

---

Those articles of food which contain the largest percentage of nitrates are the best builders of the muscular system; phosphates are most needed for the nerves and brain; carbonates help to form the fat. Beans, peas, oats, salmon, eggs and beef all contain plenty of nitrates; the same foods, and, in addition, codfish, contain an abundance of phosphates. Butter, rice, cabbage, corn, and beans provide the carbonates. Eggs fried in butter, or codfish similarly treated, constitute a perfect flesh-former.

